# How depression and ADHD relate to exercise addiction: a cross-sectional study among frequent exercisers

**DOI:** 10.3389/fpsyg.2024.1427514

**Published:** 2024-09-25

**Authors:** Lavinia Baltes-Flueckiger, Aline Wagner, Isabel Sattler, Maximilian Meyer, Amos Tschopp, Marc Walter, Flora Colledge

**Affiliations:** ^1^Psychiatric and Psychotherapeutic Clinic, Psychiatric Services Aargau, Windisch, Switzerland; ^2^University of Basel, Basel, Switzerland; ^3^Department of Sport, Exercise and Health, University of Basel, Basel, Switzerland; ^4^Psychiatric University Clinics Basel, University of Basel, Basel, Switzerland; ^5^Faculty of Health Sciences and Medicine, University of Lucerne, Lucerne, Switzerland

**Keywords:** exercise addiction, depression, ADHD, hours of exercise, cross-sectional study

## Abstract

**Background:**

To date, there are no official diagnostic criteria for the frequently reported phenomenon of exercise addiction. Therefore, the aim of the present study was to investigate how mental disorders, specifically depression and attention-deficit hyperactivity disorder (ADHD), are related to exercise addiction (EA).

**Methods:**

A total of 173 participants aged between 18 and 70 years, who reported exercising more than 10 h a week and continued to exercise despite injury or illness, answered questionnaires including the Exercise Dependence Scale, the Beck Depression Inventory, and the Homburger ADHD scale for adults. Multiple linear regression analyses were performed adjusting for relevant confounders (age, gender) and stepwise regression was used to identify which of the two mental disorders is the more influential predictor of EA.

**Results:**

Pearson correlation analysis showed that depressive symptoms [r (171) = 0.422, *p* < 0.00] and ADHD symptoms [r (171) = 0.308, *p* < 0.001] were positively correlated with EA symptoms. The relation between depressive symptoms and EA remained after adjusting for confounders in the regression model (*B = 20.531; t(170) = 5.950; 95% CI [13.719, 27.343]; p < 0.001*). Similarly, the positive link between ADHD symptoms and EA persisted after controlling for confounders (*B = 15.507; t(170) = 3.771; 95% CI [7.389, 23.625]; p < 0.001*). Additionally, a stepwise regression model identified that depressive symptoms are a stronger predictor for EA than ADHD symptoms.

**Conclusion:**

Depressive symptoms seem to be a stronger predictor for EA compared to ADHD symptoms in frequent exercisers. Although individuals with ADHD May exercise extensively, they might be less at risk for EA than individuals with depression. These results contribute to the complex characterization of the psychiatric profile of individuals with exercise addiction, and underline the need for further research elucidating the interplay between mental disorders and EA.

## Introduction

Exercise addiction (EA) is a physically and psychologically pathological phenomenon and was first described in Baekeland (1970). EA is characterized as a rigid engagement in physical exercise despite its adverse psychological, physical or social consequences (e.g., neglecting friends, family, and professional obligations), recognition of these negative consequences, the loss of control and withdrawal symptoms when reducing or stopping physical exercise (Szabo et al., 2015; Freimuth et al., 2011). These characteristics are in line with the symptoms of substance use disorders (Weinstein and Weinstein, 2014) as well as the six common components of behavioral addictions, e.g., salience, mood modification, tolerance, withdrawal, conflict, and relapse (Griffiths, 2005). However, to date EA, like other posited addictive disorders akin gambling disorder, is not classified as a behavioral addiction in any of the international classifications of mental disorders due to insufficient evidence for being a mental dysfunction (Egorov and Szabo, 2013; Macfarlane and Owens, 2016; Potenza, 2014).

Substance use disorders and addictive disorders have been shown to be accompanied by other co-ocurring mental disorders including depression, anxiety, attention-deficit hyperactivity disorder (ADHD), and post-traumatic stress disorder (Lai et al., 2015; Emmerik-van et al., 2012). Mood disorders are the most common psychiatric comorbidities among individuals with substance use disorders (Quello et al., 2005; Kessler et al., 1997). An epidemiological study showed that individuals with depression were approximately twice as likely to have a substance use disorder compared to individuals without any mood disorder (Kessler et al., 1997). Among individuals seeking treatment for any substance use disorder, an estimated 60% had experienced at least one mood disorder, and 42% an anxiety disorder (Grant et al., 2004).

Besides mood disorders, ADHD is another common psychiatric comorbidity among individuals with substance use disorder. ADHD in childhood is a stable predictor for substance use in adulthood (Dirks et al., 2017) and evidence suggested that ADHD is associated with an earlier onset and an increased risk for the development of substance use disorder (Wilens et al., 2011; Biederman et al., 1995; Young et al., 2015). A study with Swedish twins (*n* = 18′167) showed that increased ADHD symptoms were related with increased odds for all substance use disorders, specifically 2.54 for multiple drug use and 3.58 for alcohol dependence (Johansson Capusan et al., 2019). A meta-analysis and meta-regression analysis reported that 23% of individuals with substance use disorder met DSM criteria for comorbid ADHD (Emmerik-van et al., 2012).

Whereas the association between mental disorders and substance use disorders is well established, there is limited evidence on co-occurring mental disorders in exercise addiction. A recent review of twenty studies has concluded that individuals at risk for exercise addiction show a broad range of comorbid mental disorders (Colledge et al., 2020). However, the majority of these studies has focused on eating disorders, suggesting a positive association between eating disorder symptoms and EA (Colledge et al., 2020; Alcaraz-Ibáñez et al., 2020; Trott et al., 2021; Gori et al., 2021). A study with regular exercisers suggested that bulimia, drive for thinness and body dissatisfaction are a risk factor for EA. Evidence addressing other mental disorders such as depression, anxiety and ADHD in individuals with EA is scarce and results are mixed.

Two studies have shown that depressive disorders were highly prevalent (52–56%) among individuals with exercise addiction (Meyer et al., 2021; Tschopp et al., 2023). Further studies have found that higher depression scores and anxiety scores were associated with exercise addiction in recreational exercisers (Alcaraz-Ibáñez et al., 2022; Costa et al., 2013; Li et al., 2015; Weinstein et al., 2015; Lukács et al., 2019). This link could not be replicated in other studies with recreational exercisers (Lukács et al., 2019; Jee and Eun, 2018; Levit et al., 2018) nor in professional exercisers (Levit et al., 2018; Mayolas-Pi et al., 2016). In contrast, it is well established that exercise can significantly reduce depressive symptoms and exercise is recognized as evidence-based treatment for depression (Schuch et al., 2016).

Concerning ADHD, currently only two studies investigated the association between ADHD and EA. Whereas individuals with childhood ADHD showed higher prevalence of excessive exercising compared to individuals without ADHD, this result was not established in adult ADHD (Berger et al., 2014). In contrast, Colledge et al. (2022) showed that individuals at risk for EA had higher scores for adult ADHD.

In summary, individuals with substance use disorders often suffer from a mental disorder such as depression, anxiety, or ADHD. This psychiatric comorbidity appears to be also present in addictive behaviors, but evidence in EA is still in its infancy. Since depression and ADHD are two common comorbidities among individuals with substance use disorders, it is crucial to investigate these mental disorders in EA. Existing research on the link between depression and ADHD in EA is limited and has shown inconclusive results. Additionally, the direction of exercise addiction and comorbid mental disorders remains unclear (Weinstein and Szabo, 2023). To better characterize the psychiatric profile of individuals with exercise addiction, further research on the association between ADHD and depression with EA is paramount. Therefore, the aim of the present study was to evaluate whether depression and ADHD symptoms May predict EA symptoms controlling for potential confounders in frequent exercisers. Additionally, we investigated which one of the two mental disorders is more strongly associated with EA.

## Materials and methods

### Participants

Recruitment was conducted between 2019 and 2021. Potential participants were recruited via flyers in gyms, physiotherapy centers, sports clubs, pharmacies and public transport and via internet advertisement on the University of Basel public forum. Inclusion criteria were age between 18 and 75 years, exercise of more than 10 h per week, continuing to exercise despite injury or illness, and fluency in German. Exclusion criteria were regular weeks with fewer than 10 h of exercise and exercising only with a mild cold, but stopping exercise for anything more severe. These criteria were selected as they May be indicative of a potential behavioral addiction in line with DSM-5 criteria for gambling disorder (e.g., continuance, tolerance).

Interested persons contacted the study team via email or telephone call. Eligible participants were then invited to provide written informed consent, and to complete the first phase of the study including a set of questionnaires. Prior to the outbreak of COVID-19, questionnaires were provided in paper form. Following the COVID-19 measures, questionnaires were completed in electronic form. Some results of this study have already been published focusing on (Meyer et al., 2021; Tschopp et al., 2023; Colledge et al., 2022). Two subsequent phases, a clinical interview and FMRI, are ongoing and results of these phases will be reported in future publications. Participants received CHF 40 for the first examination and an additional CHF 150 for taking part in the further phases of the study. The study is in line with the Declaration of Helsinki, and was approved by the local ethics committee “Ethikkommission Nordwest- und Zentralschweiz.”

### Measures

Exercise addiction was assessed with the Exercise Dependence Scale (EDS). The EDS is a validated 21-item questionnaire which was developed in line with the DSM-IV Criteria for substance use disorders (Hausenblas and Downs, 2002). EDS includes seven subscales (tolerance, withdrawal, intention effects, loss of control, time spent in the behavior, conflict with other activities and continuance) and items were rated on a six-point Likert scale. Scores range from 21 to 126 with higher scores indicating more severe symptoms of exercise addiction. A score of 15 or more on at least three of the seven subscales indicates that respondent is at risk for exercise addiction. A score between 9 and 14 on at least three subscales indicates the respondent is symptomatic but not at risk, and scores below 9 are classed as non-symptomatic. The German translation of the EDS has been shown to have good psychometric properties (Müller et al., 2014).

Depressive symptoms were measured with the Beck Depression Inventory (BDI) (Beck et al., 1961). The BDI consists of 21 items and showed adequate validity and reliability (Beck et al., 1988). The score ranges from 0 to 63 with higher scores indicating more severe depressive symptoms(cut-off for mild to moderate depression ≥10).

The ADHD self-rating scale of the Homburger ADHD Scale for Adults (HASE) was used to assess ADHD symptoms. HASE includes 22 items that are rated on a four-point Likert scale with higher scores indicating more severe ADHD symptoms. Authors suggest a cut-off of 15 for ADHD. HASE showed satisfactory psychometric properties (Schmidt and Petermann, 2008).

Sociodemographic data were assessed including gender, age, education and marital status. In addition, participants reported the number of hours per week they spend exercising, the types of exercise they did, and whether they took part in competitions. All data were self-reported.

### Statistical analysis

To examine the association between mental disorders and EA, multiple linear regressions were computed. Scores of depressive symptoms (BDI) and ADHD symptoms (HASE) were the independent variables. Each score was the sum of the single items of the respective scale. Symptoms of EA (mean score of EDS) served as the dependent variable. Pearson correlations were initially used to assess the relationships between EA, depressive (BDI), and ADHD (HASE) symptoms. Subsequently, by means of linear regressions, these two correlations were adjusted for age and gender. Gender was dummy coded with the category ‘male’ as the reference, respectively. Additionally, a stepwise regression analysis was performed to identify which of the two mental disorders—BDI and HASE - is the more influential predictor of the outcome EA.

The assumptions for linear regression were tested. Variance inflation factors (VIF) studying multicollinearity of the model were not problematic. To meet the assumption of the normal distribution of residuals, scores of depressive symptoms and ADHD symptoms were logarithmically calculated for inferential statistics. *p*-values were 2-sided, and statistical significance was set at *α* = 0.05. Analyses were conducted with R software version 1.2.5033 and SPSS version 29.0.

## Results

A total of 173 participants completed the questionnaires and were included in the analyses. Forty one participants (24%) had a score of 15 or more on at least three of the seven subscales of the EDS and thus, were found to be at risk for EA. Sample characteristics are shown in Table 1.

**Table 1:** Sample characteristics (*n* = 173).

Gender (*n* = 172, %)	
Female	65 (38%)
Male	107 (62%)
Age in years (mean, SD)	30.6 (13.3)
Education (*n* = 156, %)	
High School	27 (16%)
Diploma	80 (46%)
University	49 (28%)
Marital status (*n* = 153, %)	
Single	123 (71%)
Married	25 (15%)
Divorced	5 (3%)
Depressive symptoms (mean, SD)	7.3 (7.2)
ADHD symptoms (mean, SD)	14.9 (9.9)
Exercise addiction symptoms (mean, SD)	74.1 (19.1)
Hours of exercise per week (mean, SD)	12.6 (4.8)
Type of exercise (*n* = 172, %)	
Endurance training	62 (36%)
Weight training	46 (27%)
Play sports	30 (17%)
Martial arts	20 (12%)
Mountaineering	8 (5%)
Acrobatics, Dance	6 (4%)
Competition (yes; *n*, %)	96 (55%)

Number of participants for whom data were available for each measure are given where different from the total for the group. ADHD = Attention-deficit hyperactivity disorder; SD = Standard deviation.

Pearson’s correlation analysis revealed that EDS score was positively correlated with BDI score (r (171) = 0.422, *p* < 0.001) and ADHD symptoms (r (171) = 0.308, *p* < 0.001). These significant relationships persisted in regression models after adjustment for gender and age. Depressive symptoms were significantly associated with EA symptoms adjusted for gender and age (*B* = 20.531; t(170) = 5.950; 95% CI [13.719, 27.343]; *p* < 0.001). The model was satisfactory (*F*(3,168) = 15.926, p < 0.001, R^2 =^ 0.221, Adjusted R^2 =^ 0.208). Similarly, the relation between ADHD symptoms and EA symptoms persisted after including the covariates (*B* = 15.507; t(170) = 3.771; 95% CI [7.389, 23.625]; *p* < 0.001) with a significant overall model fit (F(3,168) = 8.436, *p* < 0.001, R^2 =^ 0.131, Adjusted R^2 =^ 0.115). For both multiple regression models, VIF scores were not problematic for all independent variables, indicating no multicollinearity concern (Tables 2, 3). Detailed results from the regression models are presented for depressive symptoms in Table 2 and for ADHD symptoms in Table 3.

**Table 2:** Results of regression analysis between depressive symptoms and exercise addiction symptoms adjusted for age and gender.

	Unstandardized coefficients		Standardized coefficients				Collinearity statistics	
	*B*	*SE*	*β*	*t*	95% CI for *B*	*p*-value	Tolerance	VIF
Depressive symptoms	20.531	3.451	0.411	5.950	13.719, 27.343	<0.001	0.970	1.031
Gender	2.173	2.703	0.056	0.804	−3.163, 7.508	0.423	0.969	1.032
Age	−0.278	0.097	−0.195	−2.855	−0.470, −0.086	0.005	0.998	1.002

95% CI = 95% Confidence interval; SE = Standard error; VIF = Variance inflation factor.

**Table 3:** Results of regression analysis between ADHD symptoms and exercise addiction symptoms adjusted for age and gender.

	Unstandardized coefficients		Standardized coefficients				Collinearity statistics	
	*B*	*SE*	*β*	*t*	95% CI for *B*	*p*-value	Tolerance	VIF
ADHD symptoms	15.507	4.112	0.285	3.771	7.389, 23.625	<0.001	0.905	1.105
Gender	6.075	2.829	0.155	2.147	0.490, 11.660	0.033	0.987	1.013
Age	−0.164	0.108	−0.115	−1.521	−0.376, 0.049	0.130	0.911	1.097

ADHD = Attention-deficit hyperactivity disorder; 95% CI = 95% Confidence interval; SE = Standard error; VIF = Variance inflation factor.

Stepwise regression showed a positive relation between EA symptoms with both—depressive symptoms and ADHD symptoms. In the first step, BDI was entered into the model, resulting in a significant improvement, *F*(1,171) = 36.97, p < 0.001 (R^2^ = 0.178, adjusted R^2^ = 0.173). In the second step, HASE was added, leading to another significant enhancement, *F*(2,170) = 23.36, *p* < 0.001 (R^2^ = 0.216, adjusted R^2^ = 0.206). Details on each step are presented in Table 4.

**Table 4:** Multiple stepwise regression analyses between depressive and ADHD symptoms with exercise addiction symptoms.

	Unstandardized coefficients		Standardized coefficients				Collinearity statistics	
	*B*	*SE*	*β*	*t*	*p-*value	95% CI for B	Tolerance	VIF
Model 1								
(Constant)	57.890	2.981		35.906	<0.001	52.006, 63.775		
Depressive symptoms	21.259	3.496	0.422	6.881	<0.001	14.357, 28.161	1.000	1.000
Model 2								
(Constant)	47.960	4.535		10.577	<0.001	39.009, 56.911		
Depressive symptoms	18.285	3.579	0.363	5.108	<0.001	11.219, 25.350	0.916	1.092
ADHD symptoms	11.148	3.894	0.203	2.863	0.005	3.460, 18.835	0.916	1.092

ADHD = Attention-deficit hyperactivity disorder; 95% CI = 95% Confidence interval; SE = Standard error; VIF = Variance inflation factor.

## Discussion

The current study shed further light on the relation between mental disorders and EA. In particular, results showed that depressive and ADHD symptoms were positively associated with EA symptoms in frequent exercisers. Furthermore, the relation between depressive and ADHD symptoms with EA symptoms remained after adjusting for confounders (age and gender). Additionally, we showed that depressive symptoms appeared to be a stronger predictor for EA symptoms than ADHD symptoms. This finding indicates that depressive symptoms play a stronger role in EA compared to ADHD symptoms. The divergence in associations across different mental disorders highlights the need for a nuanced understanding of the factors influencing EA that need to be explored in further studies.

### Depression and exercise addiction

Our results are consistent with previous studies that reported a positive association between depression and EA in recreational exercisers (Alcaraz-Ibáñez et al., 2022; Costa et al., 2013; Li et al., 2015; Weinstein et al., 2015), while it contrasts the null finding in professionals (Levit et al., 2018; Mayolas-Pi et al., 2016). There is broad evidence that exercise May have antidepressant effects (Schuch et al., 2016). Therefore, individuals with depressive symptoms May adopt exercise as a coping strategy to alleviate negative emotions. In certain individuals, it is posited that this May lead to exercise behavior being used as a maladaptive coping mechanism (Starcevic and Khazaal, 2017). Thus, depressive symptoms might be a potential risk factor for EA in specific individuals, a factor which deserves increased attention, given the widespread prescription of exercise for individuals with depressive disorders. Therefore, the use of exercise as treatment for depression should be supervised. Additionally, depressive symptoms May also be an adverse psychological effect of EA. Cosh and colleagues, who also identified high levels of depressive symptoms in compulsive exercisers, further emphasize the need to address comorbid disorders in order to ensure successful remission from unhealthy exercising behavior (Cosh et al., 2023). Consequently, a recommendation to screen for depressive symptoms in individuals reporting EA May be warranted. Importantly, longitudinal studies are needed to elucidate the bidirectional association between depression and EA.

### ADHD and exercise addiction

The present study showed that also the association between ADHD symptoms and EA persist after adjusting for confounders. Our results are partly in line with previous findings. To date, only one study has reported a co-occurrence of ADHD and EA. Whereas Berger and colleagues showed a link between childhood ADHD, they did not establish an association between adult ADHD and EA (Berger et al., 2014). However, there is highly suggestive evidence that exercise is associated with improvement of inhibitory control, inattention, and cognitive flexibility in adolescents (Dastamooz et al., 2023) and adults with ADHD (Den Heijer et al., 2017). In a review of the potential benefits and risks of exercising, Caponnetto and colleagues (Caponnetto et al., 2021) also emphasize that exercise appears to be protective against ADHD symptoms. Similar to depression, individuals with ADHD May use exercise as a coping strategy to handle their ADHD symptoms, e.g., to improve executive functions or reduce impulsitivity (Pontifex et al., 2013). However, although individuals with ADHD May exercise extensively, they May be less at risk for EA than individuals with depression as our result showed that depressive symptoms showed a stronger relation with EA compared to ADHD symptoms. Additionally, the different subtypes of ADHD (inattentive type, hyperactive/impulsive type, and combined type) May be differently associated with EA. One study with Swedish twins (*N* = 18′167) reported that ADHD symptoms and subtypes were associated with increased risk for substance use disorders, with no difference between ADHD subtypes (Johansson Capusan et al., 2019). Research is needed to elucidate this complex interaction between ADHD subtypes in EA to improve our understanding of the nuanced psychiatric profile of individuals with EA.

## Limitations

This study has limitations. The cross-sectional study design calls for cautious interpretation of our findings. It remains unclear whether comorbid mental disorders May contribute to the development of EA, EA May be maintained by them, or EA May arise as a result of mental disorders. Longitudinal studies are needed in order to draw inferences on the direction of the relation between mental disorders and EA (Weinstein and Szabo, 2023). Furthermore, all data are based on self-reported measures. It has been shown that self-report questionnaires tend to lead to prevalence overestimates in EA (Szabo, 2018). Moreover, although depression and ADHD were assessed with validated self-report measures, future research should use official international classifications to diagnose depression and ADHD. Furthermore, in this article we focused on the association of depression and ADHD with EA. However, further mental disorders might also play an important role in EA. Specifically, anxiety is of special interest as anxiety and depression often occur comorbidly (Kessler et al., 1996). Therefore, future research should investigate a broader range of mental disorders in EA. In addition, the study was conducted during the outbreak of COVID-19. In Switzerland, several restrictions were implemented such as closing of gyms. This circumstance might have confounded the present relations between mental disorders and EA. For example, the prevalence of EA during the first lockdown in Italy appeared to be higher than in previous studies (Ceci et al., 2023). It should also be noted that this study does not include a control group of non-exercising individuals – consequently, differences in depression rates between exercisers and non-exercisers cannot be extrapolated here.

## Conclusion

The current study adds to the existing body of literature by providing empirical evidence in support of the association between depressive and ADHD symptoms with EA, irrespective of gender and age. Depressive symptoms appeared to be more strongly related with EA than ADHD symptoms. Our findings underline the importance of further investigating the temporal interplay between comorbid mental disorders and EA. Simultaneously, our results contribute to the important evidence characterizing the psychiatric profile of EA and are a further step toward classifying EA as an official diagnosis.

## Data Availability

The raw data supporting the conclusions of this article will be made available by the authors, without undue reservation.
